# Specific Immunotherapy in a Murine Model of Grass Pollen (Phl p5b)-Induced Airway Inflammation

**DOI:** 10.3389/falgy.2021.777545

**Published:** 2021-12-16

**Authors:** Matthias Stiehm, Marcus Peters

**Affiliations:** ^1^Department of Experimental Pneumology, Ruhr-University Bochum, Bochum, Germany; ^2^Department of Molecular Immunology, Ruhr-University Bochum, Bochum, Germany

**Keywords:** allergen specific immunotherapy, murine model, *Phleum pratense*, allergic asthma, sensitization

## Abstract

**Background:** The use of ovalbumin as a model allergen in murine models of allergic asthma is controversially discussed since it is not an aeroallergen and sensitization can only be achieved by using strong Th2-inducing adjuvants. Therefore, in this study, a murine model of asthma has been established in which sensitization against the major grass pollen allergen Phl p5b was performed without using aluminum hydroxide (alum). We used this model for specific immunotherapy.

**Methods:** Female, 5–6-week-old mice were sensitized by six subcutaneous (s.c.) injections of 20 μg Phl p5b followed by four provocations to induce allergic airway inflammation. For desensitization, 1 mg of Phl p5b was injected subcutaneously during allergen challenge for one to a maximum of four times. Three days after the last challenge, the allergic immune response was analyzed.

**Results:** Sensitized and challenged animals showed a significant infiltration of eosinophils into the airways, and the production of interleukin-5 (IL-5) by *in vitro* re-stimulated splenocytes could be detected. Furthermore, hyper-responsiveness of the airways was verified by invasive measurement of airway resistance in methacholine-challenged animals. Desensitized animals showed a significant reduction of all parameters.

**Conclusion:** In this study, a murine model of asthma has successfully been established by sensitization against the clinically relevant allergen Phl p5b without using alum. S.c. injection of allergen dose dependently led to desensitization of sensitized mice. We suggest that this model is useful to study adjuvant effects of immune modulatory substances on immunotherapy without the interference of alum.

## Introduction

There were different endotypes of asthma described in the literature (1). The most common one is the atopic endotype with eosinophilic inflammation. However, also non-atopic endotypes were described often going along with neutrophilic inflammation (2). Moreover, non-atopic endotypes with eosinophilic inflammation were described in the literature (3). To date, the use of ovalbumin (OVA) in Balb/c mice to induce the symptoms of allergic asthma, such as airway hyper-reactivity, eosinophilia in lung tissue, systemic allergen-specific immunoglobulin E (IgE), and increased Th2 cytokine production, is a well-accepted model in asthma research. Immunotherapeutic studies in this model by subcutaneous (s.c.) injection of high allergen doses have also successfully been performed (4). However, the use of OVA as a model allergen is controversially discussed for several reasons. As sensitization against OVA only succeeds by using strong TH2-inducing adjuvants, such as aluminum hydroxide (alum) and since OVA, is not an allergen relevant for the respiratory tract, this model bears relatively little relation to allergic airway disease in humans. Furthermore, the immunomodulatory effects of alum might also influence the allergic immune response since it could be shown that alum alone already causes an increase in IgE levels (5). Moreover, it is an activator for several other immune pathways, such as the activation of the inflammasome (6). Thus, data derived from OVA-alum experiments are influenced by unknown immunomodulatory effects caused by different adjuvant substances.

Other adjuvants, such as pathogen-associated molecular patterns, like lipopolysaccharide (LPS), have also been reported to enhance allergic sensitization. It has been shown that the commonly used grade V OVA from Sigma contains biological relevant doses of endotoxins (7). Its influence on sensitization in the OVA model is dose dependent (8). There is an urgent need for mouse models of asthma with clinically relevant allergens, e.g., by sensitizing mice toward house dust mites, Alternaria, or Ragweed (9). However, most of these established models use extracts of allergens that contain unknown amounts of pathogen-associated molecular patterns. These may act in an adjuvant manner, thereby modifying the natural mechanism of sensitization to an unknown degree. Consequently, it is important to use an allergen with a well-characterized amount of adjuvant substances. Not only the type of adjuvant but also the amount given can have an influence on the polarization of the T cell response (10).

In this study, we used recombinant Phl p5b for sensitization to develop a murine model that is useful in studies of hypo-sensitization. Mice were sensitized against the clinically relevant recombinant grass pollen allergen Phl p5b (c-terminus) without using alum. Immunotherapy was administered during the challenge phase since allergic patients might also be naturally exposed to an allergen during their therapy. To demonstrate the functionality of this model and the efficacy of immunotherapy, airway hyper-reactivity, inflammatory markers, and different antibodies produced in response to the allergen were analyzed.

## Methods

### Animals

Females, 5–6-week-old Balb/c, mice were purchased from Charles River, Sulzfeld, Germany. Mice were acclimated to the animal facility for 1 week prior to experiments. Food and water were provided *ad libitum*. All animal experiments were approved by the appropriate governmental authority (Landesamt für Natur, Umwelt und Verbraucherschutz Nordrhein-Westfalen, Germany).

### Allergen-Preparation

Phl p5b (C-terminus) was recombinantly expressed in *Escherichia coli* K12 DH5α, transformed with pProExHTa (11). Briefly, bacterial cultures were grown to an optical density of 0.6–1.0 (OD 600 nm) at 37°C. Transcription was induced by Isopropyl β-d-1-thiogalactopyranoside (IPTG). After 4 h of transcription, bacteria were lysed by ultrasonification and centrifugated to remove insoluble components. His-tagged Phl p5b was isolated by His-Gravitrap columns. Endotoxin contaminations were removed by using EndoTrap-blue columns (Profos AG, Regensburg, Germany) according to the instructions of manufacturers. Afterward Phl p5b was lyophilized and stored at −20°C until reconstitution. Residual endotoxin was measured with the chromogenic kinetic limulus amebocyte lysate (LAL test) assay as described before (12).

Allergen solutions were filtered through a 0.22 μm filter, and the concentration of allergen was determined by UV-photometrical methods prior to each application.

### Sensitization, Airway Challenge, and Immunotherapy

**Figure 1:**
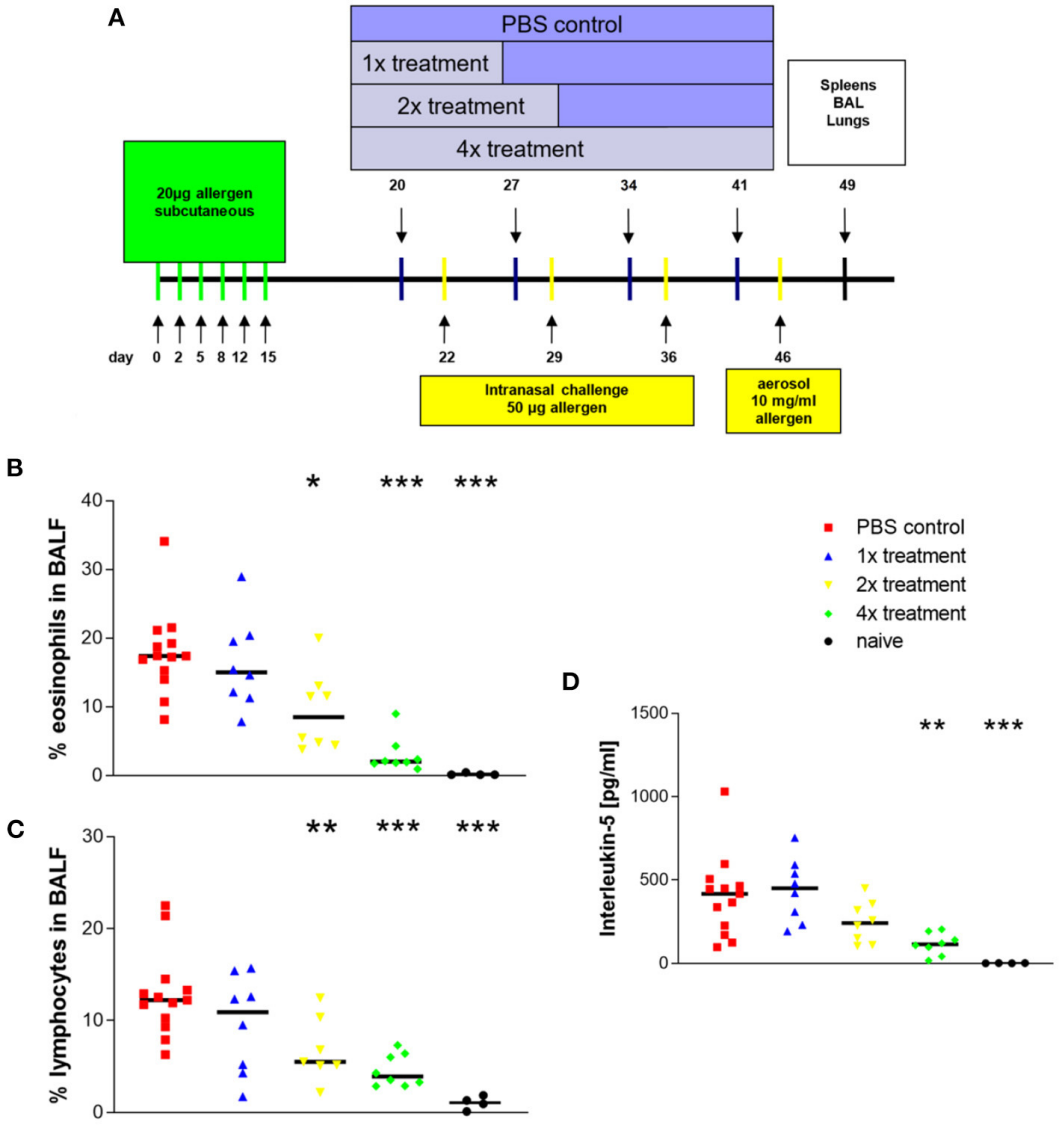
Eosinophilic airway inflammation that is induced with Phl p5b is reduced by allergen-specific immunotherapy. Design of *in vivo* experiments **(A)** Systemic sensitization was done by six s.c. injections of 20 μg allergen. A maximum of four s.c. injections of 1 mg allergen were given in an interval of 1 week. Challenges were done by three i.n. applications of 50 μg allergen dissolved in 50 μl phosphate buffered saline (PBS) each 1 week apart. The last challenge was performed by the aerosol challenge (10 mg/ml) via the airways. For analysis of the allergic immune response, splenocytes and bronchoalveolar lavage fluids and lungs were harvested on day 49. **(B)** Effect of treatment on the ratio of eosinophils in bronchoalveolar lavage fluids. **(C)** Effect of treatment on the ratio of lymphocytes in bronchoalveolar lavage fluids. **(D)** Effect of treatment on cytokine production of *in vitro* restimulated splenocytes (10^7^ cells). Phl p5b was used in a final concentration of 100 μg/ml. The increasing number of treatment of mice by immunotherapy results in decreasing IL-5 production by restimulated splenocytes. Naïve mice were untreated. *n* = 4–13 per group, *p < 0.05, **p < 0.01, ***p < 0.001 compared to PBS-treated control group, determined by unpaired Mann-Whitney test. s.c., subcutaneous; i.n., intranasal.

The sensitization, challenge, and immunotherapy protocol are shown in Figure 1A. Since we avoided the use of adsorbents, such as alum, in our study, we supposed that intraperitoneal injection would lead to a very fast pharmacological distribution of the allergen. Therefore, we decided to sensitize the mice via s.c. route. Briefly, mice were sensitized by six s.c. injections of 20 μg recombinant Phl p5b dissolved in 200 μl phosphate buffered saline (PBS). Injections were given in an interval of 2–3 days. Five days after the last injection, mice were treated by s.c. injection of 1 mg allergen dissolved in 200 μl PBS. Immunotherapy was performed a total of four times, each 1 week apart. A number of treatments were varied from a minimum of one time to a maximum of four times in different treatment groups. The control group received sham treatment by injection of 200 μl PBS. Two days after the start of immunotherapy, mice were challenged by intranasal (i.n.) application of 50 μg allergen dissolved in 50 μl PBS for a total of three times, each 1 week apart. Before the assessment, mice were anesthetized by i.p. injections of ketamine (65 mg/kg) and rompun (13 mg/kg).

The last challenge was done with 1% Phl p5b aerosol using a PARI-Boy aerosol generator. Mice were placed in a plastic chamber (10 L) and exposed to the aerosol for 30 min.

### Measurement of Airway Responsiveness

Twenty 4 h after the last challenge, airway responsiveness was measured in anesthetized orotrachealy intubated, spontaneously breathing animals by measurement of airway resistance after provocation with methacholine aerosol (6, 12, 25 and 50 mg/ml) as described previously (13, 14). Airway reactivity is expressed as an increase of airway resistance compared to baseline level. It had previously been shown that OVA-challenged mice have reached a maximum in airway hyper-responsiveness (AHR) to methacholine 24 h after the last OVA challenge (15). Therefore, this time point was also chosen in our model for measurements. Before the assessment, mice were anesthetized with intraperitoneal injections of etomidate (total dose: 21.5–37.5 mg/kg) and fentanyl (total dose: 0.10–0.19 mg/kg) with minimal supplementations as required.

### Bronchoalveolar Lavage (BAL)

Three days after the last challenge, mice were sacrificed. BAL was performed through a 20G tracheal cannula by injection of 2 × 1 ml PBS. Afterward, lungs were filled with 1 ml of TissueTek^®^/PBS 1:1 via the tracheal cannula, and the trachea was ligated. Lungs were removed and frozen at −80°C for further analysis of lung histology.

Bronchoalveolar lavage fluids (BALFs) were centrifuged (3,000 rpm, 8 min) to separate cells from soluble components. The supernatant was frozen at −20°C for further analysis of antibody titers. Cytospin slides of BAL cells were prepared and stained with a fast staining procedure (HAEME Schnellfärbung, Labor+Technik Eberhard Lehmann, Berlin, Germany). Percentages of eosinophils, lymphocytes, neutrophils, and macrophages were determined by light microscopy. At least 300 cells per slide were counted by a blinded investigator.

### Histology of Lung Tissue

For lung histology 10 μm frozen lung slices were cut by using a cryomicrotome. Lung slices were stained with a fast staining procedure (HAEME Schnellfärbung, Labor+Technik Eberhard Lehmann, Berlin, Germany) following the instructions of the manufacturer.

Eosinophils were stained by using a mixture of diaminobenzidine and hydrogen peroxide. Briefly, 10 μm lung slices were fixed to the microscope slide by incubating for 10 min in cold acetone. After washing 1 × in PBS and 1 × in Tris-buffer (0.05 M Tris-HCl, pH 7.6), 80 μl of diaminobenzidine substrate solution (0.05 M Tris/HCl, 1 mg/ml diaminobenzidine, 0.01% H_2_0_2_) were pipetted onto the lung slices and incubated for 5 min. Finally, slides were washed 2 × with PBS and 1 × with Aqua Dest.

### Determination of Antibody Titers in BALFs

BALFs were collected as described above. Levels of Phl p5b-specific immunoglobulin G (IgG)1 and immunoglobulin A (IgA) levels in BALF were determined by the enzyme-linked immunosorbent assay (ELISA). In short, Nunc MaxiSorp plates were coated with 20 μg/ml Phl p5b and incubated at 7°C overnight and subsequently blocked with 5% low-fat milk powder (LFMP) in PBS. After incubation with diluted samples, bound Phl p5b-specific antibodies were detected by isotype-specific antibodies goat anti-mouse IgA (polyclonal, Sigma Aldrich, Seelze, Germany) and rat anti-mouse IgG1 (clone X56, BD Biosciences, Heidelberg, Germany) both conjugated to alkaline phosphatase. ELISA was developed with pNPP as substrate, measurements of absorption were done at 405 nm. Levels of total IgE were determined by BD OptEIA ELISA Kits (BD Biosciences, Heidelberg, Germany) according to the instructions of the manufacturers.

### *In vitro* Cytokine Production of Mouse Splenocytes

Three days after the last challenge, spleens were harvested. Single cell suspensions were prepared by means of mechanical disruption, and erythrocytes were lysed. Splenocytes were cultured in a concentration of 1 × 10^7^ cells/ml in tissue culture medium (RPMI 1640, 10% FCS, 100 U/ml penicillin, 100 μg/ml streptomycin, 2 mM l-glutamine, all from Biochrom, Berlin, Germany). To induce antigen-specific cytokine production, Phl p5b was added to the culture medium to a final concentration of 100 μg/ml. Cell suspensions were cultured for 48 h at 37°C and 5% CO_2_. Supernatants of cell culture were frozen at −80°C until further analysis. Levels of interleukin-5 (IL-5) and interferon-gamma (IFN-γ) were determined by BD OptEIA ELISA Kits (BD Biosciences, Heidelberg, Germany) according to the instructions of the manufacturers.

## Results

### Airway Inflammation and Cytokine Production of *in vitro* Restimulated Splenocytes

After sensitization and challenge with Phl p5b, animals developed a marked airway eosinophilia (mean ± SEM = 18 ± 2%) (Figure 1B) an important characteristic for asthma (group description: PBS control). In comparison, published data from the OVA-alum model show an about four times higher percentage of eosinophils in the airways (16).

Treatment by immunotherapy resulted in reduced airway inflammation. A significant reduction compared to sham-treated mice could be observed with two treatments, and further reduction of airway eosinophilia was achieved with four treatments. Infiltration of lymphocytes showed the same decrease (Figure 1C). Four treatments with immunotherapy caused the strongest effect. Total cell numbers in BALF revealed no significant differences between sham-treated animals and animals that received immunotherapy. The ratio of neutrophils was beyond 3% in all groups (data not shown).

Airway eosinophilia depends on the secretion of cytokines by TH2-lymphocytes. In particular, IL-5 acts as a chemoattractant for eosinophils (17) and is produced as a consequence of sensitization. Splenocytes of sensitized and challenged animals produced the largest amounts of IL-5 as a specific reaction to re-stimulation with Phl p5b (Figure 1D; mean ± SEM = 402 ± 67 pg/ml). Published data from the OVA-alum model show that the production of IL-5 of splenocytes is overall higher than observed here (14, 16). Increasing numbers of immunotherapy caused decreasing production of IL-5 by restimulated lymphocytes, thereby indicating a dose dependency for the success of immunotherapy.

Interferon-γ production of splenocytes revealed no significant differences between the different groups (data not shown), thereby, indicating that no Th1-immune response has been induced due to treatment with specific immunotherapy (SIT).

### Lung Histology

**Figure 2:**
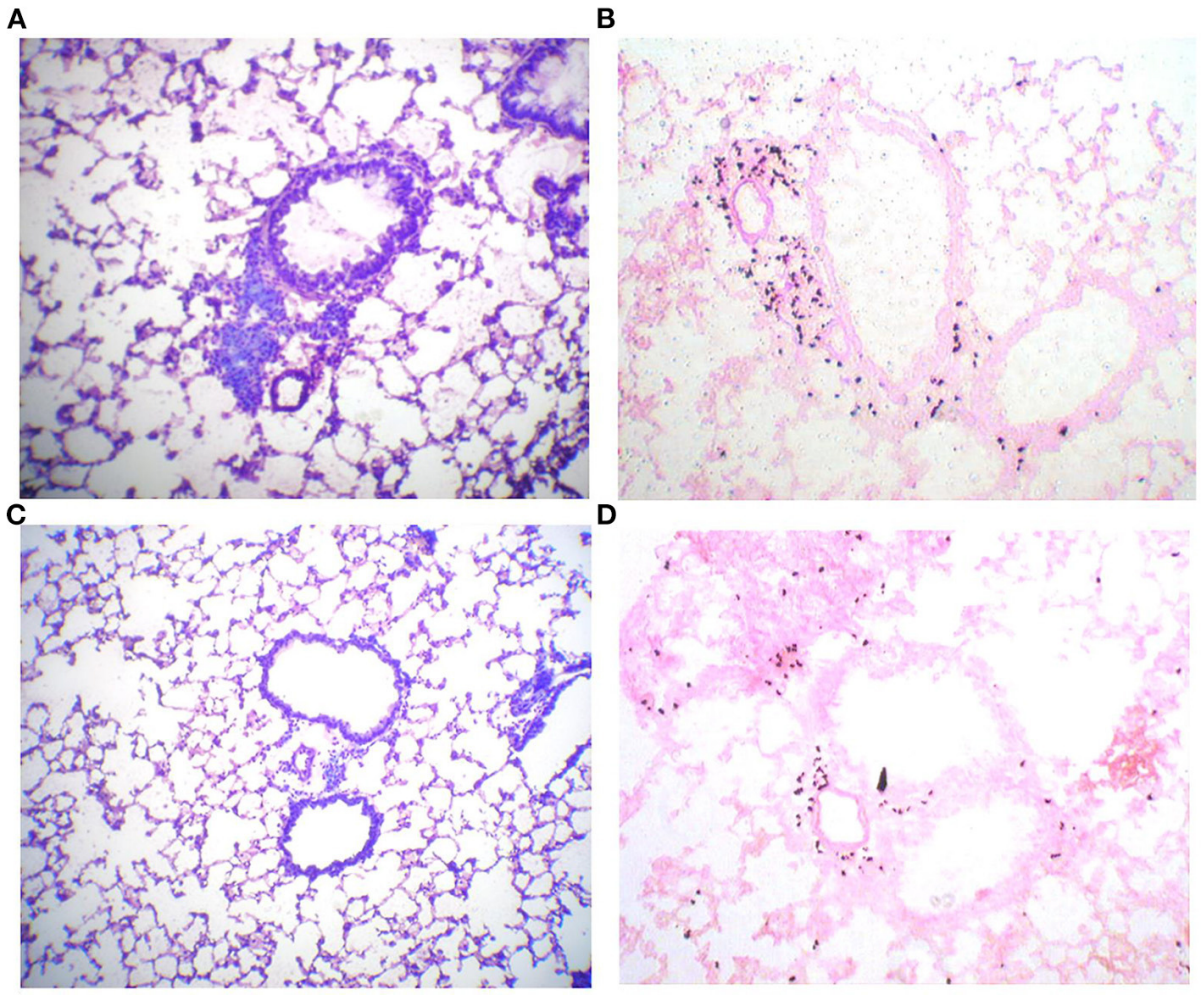
Phl p5b-induced leucocytic infiltrates of lung tissue are reduced by SCIT. Lung histology: 10 μm lung slices of a sham-treated mouse **(A,B)** and a mouse treated four times with immunotherapy **(C,D)**. **(A)** Pappenheim staining shows the accumulation of lymphocytes around broncheolus and pulmonal arteriola. **(B)** Staining of eosinophilic peroxidase with Diaminobenzidin/H_2_O_2_ reveals that these infiltrations mainly consist of eosinophils. **(C)** Pappenheim staining of a lung slice of a mouse treated with immunotherapy reveals reduced infiltration of leucocytes. **(D)** Infiltration of eosinophils is also decreased in this animal.

An important characteristic of allergen-induced airway inflammation is the accumulation of eosinophils in lung tissue. Lung slices of sensitized and challenged animals show accumulation of leucocytes around pulmonal arteriola and the broncheoli (Figure 2A). Interestingly infiltrations could only be observed directly around broncheoli, whereas no infiltrations of leucocytes into lung parenchym were detected. These infiltrations mainly consist of eosinophils, as demonstrated by eosinophil staining (Figure 2B). In contrast, infiltration of leucocytes is strongly decreased in the lungs of animals that were treated four times with immunotherapy (Figures 2C,D). However, infiltrations are still observable in treated mice, but to a much lesser extend.

### Influence of SIT on Humoral Immune Responses to Phl p5b

Since different isotypes of allergen-specific antibodies were shown to be involved in allergic immune reactions, we have measured antibodies in BAL fluid to evaluate the humoral immune response toward the allergen.

**Figure 3:**
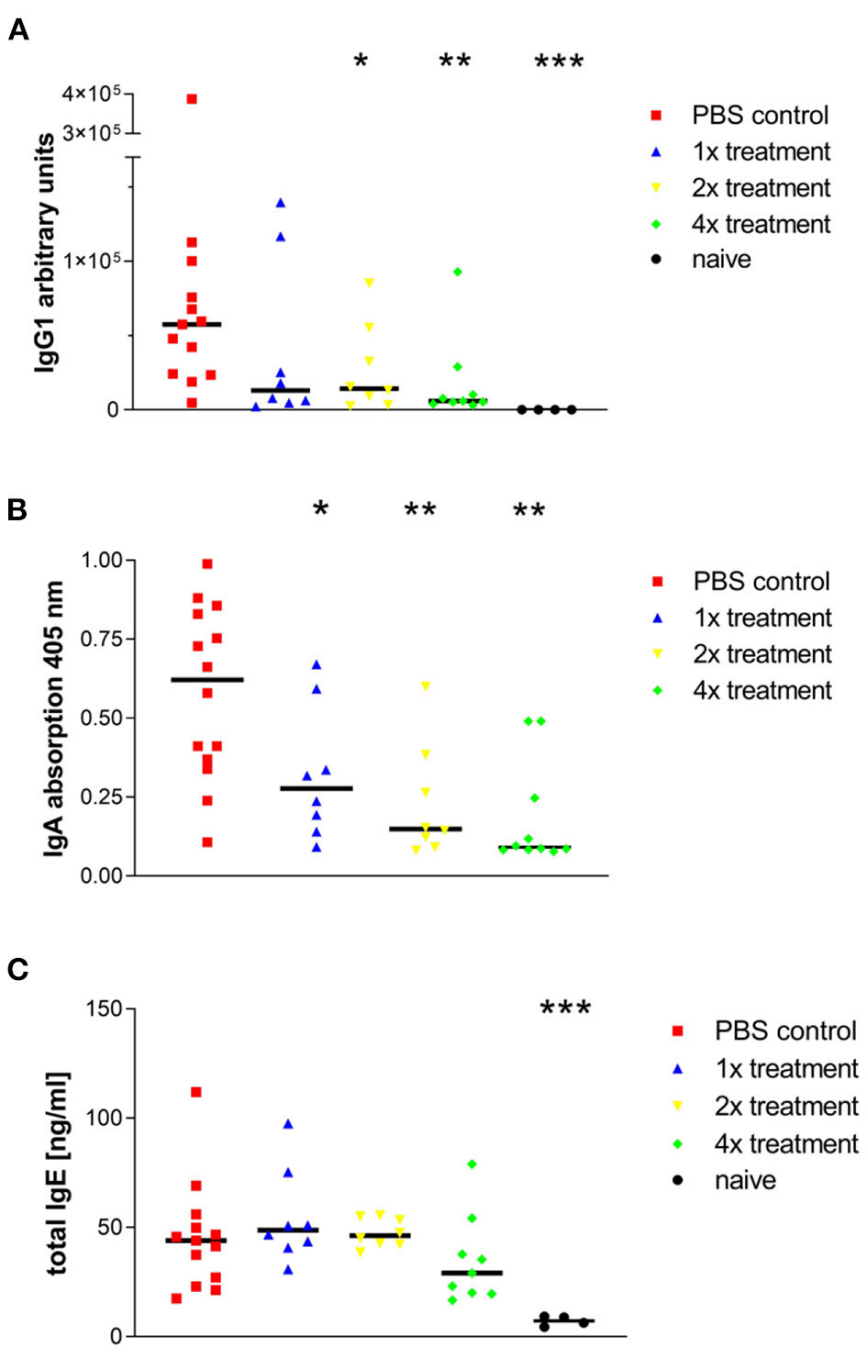
Phl p5b-induced production of IgG1, IgA, and IgE in the lung is modulated by SCIT. Measurement of antibody concentration in the airways: **(A)** Allergen-specific IgG1 titers in BALFs. The standard was a pool of sera from sensitized mice, which contained high allergen-specific IgG1 titers. Lowest dilution of standard sera was set to 100 AU. (**B)** Allergen-specific IgA titers in BALFs. Measurement of absorption at 405 nm was done in equal dilutions of BALFs (1:50). **(C)** Total IgE levels in BALFs. Four injections of 1 mg Phl p5b lead to a reduction of IgE titers in BALFs. Naïve mice were untreated. *n* = 4–13 per group,*p < 0.05, **p < 0.01 ***p < 0.001 compared to phosphate buffered saline treated control group determined by unpaired Mann-Whitney test. IgE, immunoglobulin E; IgG, immunoglobulin G; IgA, immunoglobulin A.

Sensitized and challenged animals, which did not receive SIT, produced significantly more allergen-specific IgG1 and IgA antibodies in their lungs than animals that were treated (Figures 3A,B). OVA-specific IgE could not be measured. Yet, there was an increase of total IgE in BAL fluid observed after sensitization and challenge with Phl p5b. In comparison to published reports from the OVA-alum model, the total IgE in BAL fluid was lower in our new model (14). No differences could be observed in IgE titers between sham-treated animals and mice that were treated one or two times with immunotherapy (Figure 3C). A tendency of decreased IgE production could be observed with four treatments. Furthermore, a significant decrease in IgA production could be achieved by one single treatment and for IgG1 titers by two treatments, thereby implying a strong effect of s.c. injections of allergen on humoral immune response in the lung.

### Effects of Immunotherapy on Airway Responsiveness

**Figure 4:**
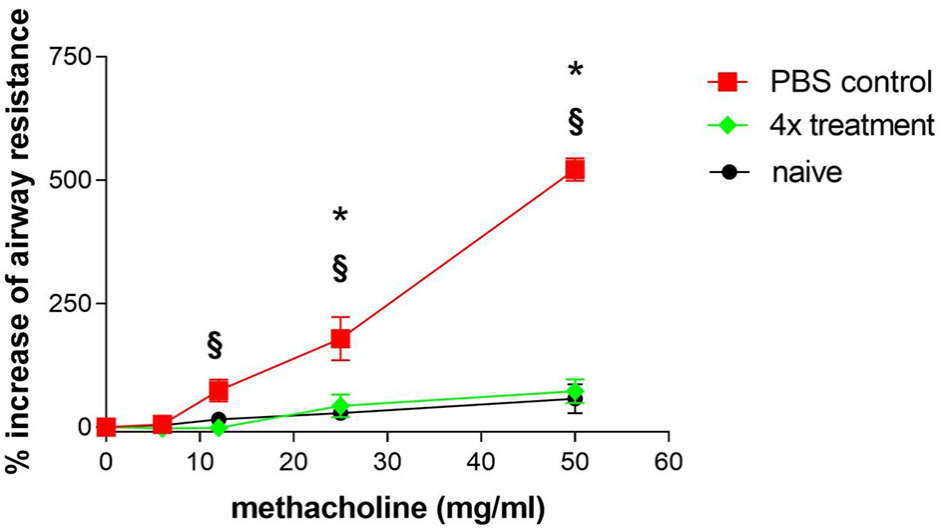
Airway hyper-reactivity as one hallmark of asthma is efficiently treated by SCIT. Airway hyper-reactivity: Measurement of airway hyper-responsiveness in sensitized and challenged mice (PBS control) and in sensitized, challenged and four times treated mice (4× treatment). Naïve mice were untreated. Airway resistance was measured in intubated spontaneously breathing mice. Each symbol represents mean ± SEM, four mice per group were measured. *p < 0.05 for 4× treatment compared to PBS control, ^§^*p* < 0.05 for naïve mice compared to PBS control, determined by unpaired Mann-Whitney test.

Another characteristic of asthma is hyper-reactivity of the airways to pharmacological, physical, chemical, and immunological stimuli. In this study, mice were stimulated with increasing concentrations of methacholine to investigate airway responsiveness. Since the method of measuring airway reactivity in orotrachealy intubated mice is laborious, this method was only used for studying the treatment group that was previously shown to be most effective. Therefore, mice that were sham-treated were compared to mice that had received four treatments with SIT. Mice that were challenged without any immunotherapy developed significant AHR that was obvious after provocation with 25 and 50 mg/ml methacholine (Figure 4). Published results in the OVA-alum model where AHR was measured with the same method showed that the increase of airway resistance was already significant after the challenge with 6 mg/ml methacholine (14).

Significant differences in airway resistance between mice that were treated four times and sham-treated mice could initially be observed at a concentration of 12 mg/ml methacholine. Mice that were treated four times with immunotherapy reached a maximum in airway resistance that was 120% above the basal level at 50 mg/ml methacholine. In contrast, sham-treated mice reached a maximum in airway resistance that was 550% above basal level. This demonstrates that immunotherapy by injection of 1 mg Phl p5b for a total of four times could reduce AHR significantly.

## Discussion

In this study, we were able to show that sensitization toward the clinical relevant grass pollen allergen Phl p5b could successfully be performed in mice without using alum as an adjuvant. Furthermore, we demonstrate that SIT by four s.c. injections of 1 mg allergen improved most of the important clinical symptoms and immunological parameters of asthma.

In our protocol, mice were treated during provocation with an allergen. We suggest that this protocol might closely mirror the natural situation, since allergic patients are similarly exposed to an allergen during their SIT.

It had previously been shown that allergen-specific immunotherapy in OVA-alum sensitized mice reduces airway inflammation and AHR (18). In our study, AHR as one of the most important characteristics of asthma has been verified in sensitized and challenged mice by exposing them to increasing concentrations of methacholine. Since the validity of the widely used whole body plethysmography (measurement of the Penh value) (19) as a meaningful measure of bronchoconstriction had been questioned by several authors (20, 21), we chose an invasive method to determine pulmonary resistance upon provocation with methacholine (13). In mice that were treated four times by specific immunotherapy, we measured a significant reduction of airway reactivity revealing that SIT improved this clinical important parameter.

Furthermore, sham-treated mice showed significant infiltration of eosinophils in lung tissue as demonstrated by lung histology and analysis of cellular composition in BALs. However, total cell numbers in BALFs of sensitized mice revealed only a slight not significant infiltration with leucocytes compared to naïve mice (data not shown). This indicates that in contrast to the OVA model inflammation is in general weaker in our model.

Splenocytes of sensitized and challenged mice produced the highest amounts of IL-5 upon *in vitro* restimulation with Phl p5b. An increasing number of immunotherapeutic treatments led to reduced production of IL-5 by splenocytes.

In contrast to other studies, which explain effects of immunotherapy by a shift from TH2 toward TH1-immune response (22), in our model we found neither increased IFN-γ production nor allergen-specific IgG2a (data not shown). This difference might be due to the fact that endotoxin contamination in the Phl p5b preparations used for this study was low (max 69 ng/mg), thereby not triggering the immune system into a Th1 direction upon immunotherapy (maximum dose of LPS per immunotherapy 69 ng). The Th1 shift observed in the OVA model might be a result of the high doses of LPS administered to the animals upon immunotherapy, since 1 mg of the commonly used Sigma Grade V OVA contains up to 10 μg of LPS.

Besides eosinophilic airway inflammation and AHR, we found increased antibody titers (IgG1, IgE, and IgA) in BALF, which were efficiently reduced by SIT. It has been shown by Peebles Jr. et al. that allergen-specific IgA titers in BAL fluid correlate with the release of eosinophilic cationic protein (ECP) and may therefore act as a marker for degranulation of eosinophils and the severity of asthma (23). Moreover, allergen-specific IgG1 titers in BAL fluids were also significantly reduced due to one single treatment. This is in contrast to the hypothesis of induction of blocking IgG1 antibodies during SIT as seen in other models (24). In our model, the decrease of IgG1 titers is associated with the success of SIT, thereby indicating that the IgG1 antibodies are not of the blocking but of the IL-4 dependent, the anaphylactic type which is able to induce degranulation of mast-cells (25).

In contrast to IgG1 titers, IgE production was low in BALFs of sham-treated mice. However, a non-significant reduction of IgE titers can be observed by four treatments with immunotherapy, indicating the success of SIT. Importantly, allergen-specific IgE titers could be measured neither systemic nor local, certainly caused by high allergen-specific IgG1 titers resulting in displacement reactions in the test system.

The interesting observation is that in our new model of eosinophilic airway inflammation eosinophilia is accompanied by relatively low levels of IgE in BALF resembles a non-atopic late-onset asthma endotype in humans (26). This endotype is associated with strong eosinophilia and enhanced corticosteroid resistance. Since models of corticoid-resistance are important research tools, in future studies it will be of interest to test for corticosteroid resistance in our new model.

The comparison with published literature (14, 16) shows that the allergic immune response is weaker in our new model compared to the commonly used OVA model. The reason for the higher IgE titers in the OVA-model is likely an artificial boost in IgE production caused by alum (5). In our model, we used allergen preparations with a maximum of 69 ng LPS/mg Phl p5b, resulting in a maximum dose of 1.4 ng LPS for each sensitization. Eisenbarth et al. reported that sensitization toward OVA could not be performed by application of 100 μg OVA contaminated with 1 ng LPS, suggesting that a certain level of LPS contamination in OVA is essential for successful sensitization (8). Although we had comparable low LPS contamination, in the Phl p5b model mice could be sensitized well, which might be due to the fact that structural features of the allergen rather than LPS contamination play a pivotal role in the sensitization process.

Taken together, we showed that sensitization against a clinically relevant allergen is possible without using alum. We suggest that this mouse model of Phl p5b induced asthma could be a helpful tool to study cellular and molecular events during allergic sensitization. In addition, this model should be useful to study the cellular and molecular processes during desensitization.

## Data Availability Statement

The original contributions presented in the study are included in the article/supplementary material, further inquiries can be directed to the corresponding author/s.

## Ethics Statement

The animal study was reviewed and approved by Landesamt für Natur, Umwelt und Verbraucherschutz Nordrhein-Westfalen, Germany.

## Author Contributions

All authors listed have made a substantial, direct, and intellectual contribution to the work and approved it for publication.

## Conflict of Interest

The authors declare that the research was conducted in the absence of any commercial or financial relationships that could be construed as a potential conflict of interest.

## Publisher's Note

All claims expressed in this article are solely those of the authors and do not necessarily represent those of their affiliated organizations, or those of the publisher, the editors and the reviewers. Any product that may be evaluated in this article, or claim that may be made by its manufacturer, is not guaranteed or endorsed by the publisher.

## References

[1] Agache I Akdis C Jutel M Virchow JC. Untangling asthma phenotypes and endotypes. Allergy. (2012) 67:835–46. 10.1111/j.1398-9995.2012.02832.x 22594878

[2] Radermecker C Louis R Bureau F Marichal T. Role of neutrophils in allergic asthma. Curr Opin Immunol. (2018) 54:28–34. 10.1016/j.coi.2018.05.006 29883877

[3] Brusselle GG Maes T Bracke KR. Eosinophils in the spotlight: eosinophilic airway inflammation in nonallergic asthma. Nat Med. (2013) 19:977–9. 10.1038/nm.3300 23921745

[4] Vissers JL van Esch BC Hofman GA Kapsenberg ML Weller FR van Oosterhout AJ. Allergen immunotherapy induces a suppressive memory response mediated by IL-10 in a mouse asthma model. J Allergy Clin Immunol. (2004) 113:1204–10. 10.1016/j.jaci.2004.02.041 15208606

[5] Seitzer U Bussler H Kullmann B Petersen A Becker W-M Ahmed J. Mouse strain specificity of the IgE response to the major allergens of *Phleum pratense*. Int Arch Allergy Immunol. (2005) 136:347–55. 10.1159/000084228 15741733

[6] Wen Y Shi Y. Alum: an old dog with new tricks. Emerg Microbes Infect. (2016) 5:e25. 10.1038/emi.2016.40 27004761 PMC4820675

[7] Watanabe J Miyazaki Y Zimmerman GA Albertine KH McIntyre TM. Endotoxin contamination of ovalbumin suppresses murine immunologic responses and development of airway hyper-reactivity. J Biol Chem. (2003) 278:42361–8. 10.1074/jbc.M307752200 12909619

[8] Eisenbarth SC Piggott DA Huleatt JW Visintin I Herrick CA Bottomly K. Lipopolysaccharide-enhanced, toll-like receptor 4-dependent T helper cell type 2 responses to inhaled antigen. J Exp Med. (2002) 196:1645–51. 10.1084/jem.20021340 12486107 PMC2196061

[9] Debeuf N Haspeslagh E van Helden M Hammad H Lambrecht BN. Mouse models of asthma. Curr Protoc Mouse Biol. (2016) 6:169–84. 10.1002/cpmo.4 27248433

[10] Peters M Dudziak K Stiehm M Bufe A. T-cell polarization depends on concentration of the danger signal used to activate dendritic cells. Immunol Cell Biol. (2010) 88:537–44. 10.1038/icb.2010.3 20125117

[11] Gehlhar K Rajashankar KR Hofmann E Betzel C Weber W Werner S. Lysine as a critical amino acid for IgE binding in Phl p 5b C terminus. Int Arch Allergy Immunol. (2006) 140:285–94. 10.1159/000093706 16735798

[12] Liebers V Stubel H Düser M Brüning T Raulf-Heimsoth M. Standardization of whole blood assay for determination of pyrogenic activity in organic dust samples. Int J Hyg Environ Health. (2009) 212:547–56. 10.1016/j.ijheh.2009.03.003 19395310

[13] Glaab T Mitzner W Braun A Ernst H Korolewitz R Hohlfeld JM. Repetitive measurements of pulmonary mechanics to inhaled cholinergic challenge in spontaneously breathing mice. J Appl Physiol. (1985). (2004) 97:1104–11. 10.1152/japplphysiol.01182.2003 15121749

[14] Peters M Kauth M Scherner O Gehlhar K Steffen I Wentker P. Arabinogalactan isolated from cowshed dust extract protects mice from allergic airway inflammation and sensitization. J Allergy Clin Immunol. (2010) 126:648–56.e1–4. 10.1016/j.jaci.2010.05.011 20621350

[15] Tomkinson A Cieslewicz G Duez C Larson KA Lee JJ Gelfand EW. Temporal association between airway hyperresponsiveness and airway eosinophilia in ovalbumin-sensitized mice. Am J Respir Crit Care Med. (2001) 163:721–30. 10.1164/ajrccm.163.3.2005010 11254531

[16] Peters M Kauth M Schwarze J Körner-Rettberg C Riedler J Nowak D. Inhalation of stable dust extract prevents allergen induced airway inflammation and hyperresponsiveness. Thorax. (2006) 61:134–9. 10.1136/thx.2005.049403 16244088 PMC2104583

[17] Schwarze J Cieslewicz G Hamelmann E Joetham A Shultz LD Lamers MC. IL-5 and eosinophils are essential for the development of airway hyperresponsiveness following acute respiratory syncytial virus infection. J Immunol. (1999) 162:2997–3004. 10.1164/ajrccm.162.2.9903057 10072551

[18] van Oosterhout AJ van Esch B Hofman G Hofstra CL van Ark I Nijkamp FP. Allergen immunotherapy inhibits airway eosinophilia and hyperresponsiveness associated with decreased IL-4 production by lymphocytes in a murine model of allergic asthma. Am J Respir Cell Mol Biol. (1998) 19:622–8. 10.1165/ajrcmb.19.4.3112m 9761759

[19] Hamelmann E Schwarze J Takeda K Oshiba A Larsen GL Irvin CG. Noninvasive measurement of airway responsiveness in allergic mice using barometric plethysmography. Am J Respir Crit Care Med. (1997) 156:766–75. 10.1164/ajrccm.156.3.9606031 9309991

[20] Lundblad LK Irvin CG Adler A Bates JH. A reevaluation of the validity of unrestrained plethysmography in mice. J Appl Physiol (1985). (2002) 93:1198–207. 10.1152/japplphysiol.00080.2002 12235015

[21] Mitzner W Tankersley C. Interpreting Penh in mice. J Appl Physiol (1985). (2003) 94:828–31. 10.1152/japplphysiol.00815.2002 12531918

[22] Durham SR Till SJ. Immunologic changes associated with allergen immunotherapy. J Allergy Clin Immunol. (1998) 102:157–64. 10.1016/S0091-6749(98)70079-X 9723654

[23] Peebles RS Liu MC Adkinson NF Lichtenstein LM Hamilton RG. Ragweed-specific antibodies in bronchoalveolar lavage fluids and serum before and after segmental lung challenge: IgE and IgA associated with eosinophil degranulation. J Allergy Clin Immunol. (1998) 101:265–73. 10.1016/S0091-6749(98)70392-6 9500761

[24] Wachholz PA Durham SR. Induction of 'blocking' IgG antibodies during immunotherapy. Clin Exp Allergy. (2003) 33:1171–4. 10.1046/j.1365-2222.2003.01765.x 12956735

[25] Faquim-Mauro EL Coffman RL Abrahamsohn IA Macedo MS. Cutting edge: mouse IgG1 antibodies comprise two functionally distinct types that are differentially regulated by IL-4 and IL-12. J Immunol. (1999) 163:3572–6. 10.1093/intimm/12.12.1733 10490948

[26] Gerday S Schleich F Henket M Guissard F Paulus V Louis R. Asthmatics with concordant eosinophilic disease classified according to their serum IgE status. Respir Med Res. (2021) 79:100797. 10.1016/j.resmer.2020.100797 33383519

